# Study protocol of the Center for Oral Health Research in Appalachia (COHRA) etiology study

**DOI:** 10.1186/1472-6831-8-18

**Published:** 2008-06-03

**Authors:** Deborah E Polk, Robert J Weyant, Richard J Crout, Daniel W McNeil, Ralph E Tarter, John G Thomas, Mary L Marazita

**Affiliations:** 1 Department of Dental Public Health and Information Management, University of Pittsburgh, School of Dental Medicine, Pittsburgh, PA, USA; 2 Department of Periodontics, West Virginia University, School of Dentistry, Morgantown, WV, USA; 3 Department of Biochemistry, West Virginia University, School of Medicine, Morgantown, WV, USA; 4 Department of Psychology, West Virginia University, Eberly College of Arts and Sciences, Morgantown, WV, USA; 5 Department of Dental Practice and Rural Health, West Virginia University, School of Dentistry, Morgantown, WV, USA; 6 Department of Pharmaceutical Sciences, University of Pittsburgh, School of Pharmacy, Pittsburgh, PA, USA; 7 Department of Pathology, West Virginia University, School of Medicine, Morgantown, WV, USA; 8 Department of Oral Biology, University of Pittsburgh, School of Dental Medicine, Pittsburgh, PA, USA; 9 Department of Human Genetics, University of Pittsburgh, Graduate School of Public Health, Pittsburgh, PA, USA; 10 Department of Psychiatry, University of Pittsburgh, School of Medicine, Pittsburgh, PA, USA

## Abstract

**Background:**

People in Appalachia experience some of the worst oral health in the United States. To develop effective intervention and prevention strategies in Appalachia, we must understand the complex relationships among the contributing factors and how they affect the etiology of oral diseases. To date, no such comprehensive analysis has been conducted. This report summarizes the characteristics of the sample and describes the protocol of a study determining contributions of individual, family, and community factors to oral diseases in Appalachian children and their relatives.

**Methods/Design:**

Families participated in a comprehensive assessment protocol involving interviews, questionnaires, a clinical oral health assessment, a microbiological assessment, and collection of DNA. The design of the study is cross-sectional.

**Conclusion:**

Due to its multilevel design and large, family-based sample, this study has the potential to greatly advance our understanding of factors that contribute to oral health in Appalachian children.

## Background

People in the Appalachian geographic region experience health, education, economic, and social disadvantages. West Virginia, the one state located entirely in Appalachia [1], ranks at or close to the bottom on most indicators of health and social welfare, including oral health. The poor quality of oral health may be the product of numerous factors, including mountainous terrain resulting in social isolation, relative absence of fluoridated water, culturally ingrained health-impairing customs, poor knowledge of health-promoting behavior, and low priority attached to dentition. Moreover, poor availability, difficult access, and reluctance to utilize oral health services, even those publicly funded, may be significant barriers to the adoption of oral health behaviors that prevent disease. Given the number of factors potentially involved, it is not surprising that ameliorating economic disadvantage alone has been shown to have only minimal impact on increasing utilization of dental services [2].

To develop effective intervention and prevention strategies in Appalachia, we must understand the complex relationships among these different factors and how they affect the etiology of oral diseases. To date, no such comprehensive analysis has been conducted. The University of Pittsburgh Center for Oral Health Research in Appalachia (COHRA) was established in 2000, in partnership with West Virginia University, to address this gap in our knowledge. An initial aim of COHRA is to determine the contributions of individual, family, and community factors to oral diseases in Appalachian children. We hypothesize that many of the risk factors associated with poor oral health across the lifespan have their origins in childhood or adolescence. Identifying the origins of these risk factors will help inform interventions that lead to long-term health benefits. To accomplish these aims, we designed a cross-sectional etiology study in which we assessed variables at several levels. We characterized the risk within families with respect to children's oral health and identified individual, family, and community factors associated with children's oral health.

## Method/Design

### Protocol Development

The COHRA etiology study protocol was developed over many months by a committee with expertise in genetics, microbiology, epidemiology, biostatistics, behavioral science, community assessment, rural health, and dentistry. The development of the clinical protocol was guided by the desire to keep the study visit to a reasonable length and to allow for comparisons of our results with other studies. Thus whenever possible, we adopted well established assessments. Additionally, when choices were available, we opted for the shortest possible protocol that met our scientific needs. Because some instruments required adaptation for use with both children and adults, we made age-appropriate modifications to some questionnaires. For very young children, questions were directed to a parent and were limited to those for which meaningful answers could be obtained.

### Recruitment and Screening of Families

#### Sampling Strategy

Our goal was to recruit a study population that represented the salient features of the social, cultural, and economic environment of rural Appalachia. We hypothesized that these features were not only important risks factors for the development of oral disease but also the principal cause of the oral health disparity. Because we did not consider it feasible to recruit a true random sample, we developed recruitment strategies to create a study population with broad representation across dimensions of income, education, geographic residence, and cultural identification with Appalachia. This approach allowed us to test hypotheses and characterize risk factors and relationships that are important and relevant to the high risk populations of rural Appalachia.

The unit of recruitment was the family (see family composition definition below). Family recruitment was conducted and all subject assessments occurred in two central West Virginia counties (Webster and Nicholas) and two western Pennsylvania counties (Washington and McKean). In addition to being representative of rural Appalachia, these sites were selected because they had the infrastructure necessary to support this study, including active community advisory boards and established ties to either the University of Pittsburgh or West Virginia University. Participants within these geographic locations were recruited by radio and newspaper announcements and flyers distributed at churches, retail sites, schools, medical facilities, daycare centers, and Head Start sites. Additional information was distributed at health fairs, county fairs, and public schools.

#### Screening of Families

Once a family member contacted the study, family eligibility was determined by a telephone screening interview that addressed family composition, demographic information, general health status, and medical issues. Everyone living in eligible households regardless of biological or legal relationship was invited to participate in the study and scheduled for a clinic visit. The individual completing the screening interview was designated as the proband.

#### Eligibility Criteria for Families

##### Family composition

A family had to have at least one parent-child pair in which the participating child was the biological child of a participating parent and was between the ages of 1 – 18.

##### Residence

The parent-child pair had to live together in the same household. Families had to have a permanent residence in the targeted recruitment area.

##### Health and Medical

Individuals with a neurological impairment, a severe physical or intellectual handicap, or psychosis were excluded from the study. Families were excluded if an adult or child who was part of the biological parent-child pair had either a reduced capacity to resist infection or a reduced ability to form blood clots. This included those individuals who had leukemia, cancer, unstable diabetes, a transplant, or a blood clotting disorder or who were taking corticosteroids, immunosuppressive therapy, or blood thinning medications, or who were HIV+.

### Examination

#### Procedures

Prior to the scheduled appointment, each family received a welcome packet that included a vial for them to provide a sample of their residential tap water for analysis of fluoride content.

At the start of the clinic visit, each family member underwent the age appropriate consent process, as approved by the University of Pittsburgh Institutional Review Board (coordinating center approval # 0207073, Pennsylvania site approval # 0506048) and West Virginia University Institutional Review Board (approval # 15620B). The research was in compliance with the Helsinki Declaration. After consent, each participant received an antibiotic prophylaxis screening and physical evaluation. The family members then rotated through the sections of the study protocol as described below. Following the completion of the study protocol, adult participants were given summary sheets describing their own dental treatment needs as well as those of their children. Referrals were also provided for participants who needed dental care who did not have dentists. Each member of the family was reimbursed for time and travel with a gift certificate to a retail store in the area.

In West Virginia, one full-time research team rotated among four clinical examination sites. In Pennsylvania, each site was assigned its own research team. The clinical data collection at each site was conducted by either a licensed dentist or a dental hygienist.

#### Antibiotic Screening

The need for antibiotic prophylaxis was determined for each participant using American Heart Association guidelines. For adult participants requiring prophylaxis, the dental portion of the examination was rescheduled until the participant could premedicate or obtain a release from his or her primary care physician permitting the clinical examination without premedication with antibiotics. For participants under age 18 who required prophylaxis, no dental procedure was done that would initiate bleeding, thus avoiding the need for antibiotics.

#### Physical Evaluation

Research staff measured the participant's height, weight, and girth. Girth was measured at the point immediately above the iliac crest.

#### Self-report Questionnaires and Interviews

Both self-report and interview data were collected. During the first two years of the baseline study, self-report data were collected via paper forms. However, concerns about slow completion times, possibly related to low literacy and the cumbersome nature of the self-report forms, led us to develop computer-based self-report questionnaires on PC tablets. The PC tablets were easy for participants to navigate through and could play an audio file of the questionnaires read aloud (through headphones) to the participants if necessary. Table 1 lists the questionnaires that were administered to each age group. Children ages 11 and older completed the questionnaires themselves; parents completed questionnaires for children 10 and younger.

**Table 1:** Questionnaires and interviews administered by age group

	Age Group			
Instrument	18+	14 – 17	11 – 13	1 – 10
Questionnaires				
SF-36 Health Survey [11]	X	X	X	
DUSI-R [12]	X	X^a^		
DUSI-R (Screening Form Only) [12]	X	X	X^a^	
Fagerstrom Test for Nicotine Dependence [13]	X	X	X	
Fagerstrom Test for Smokeless Tobacco Use	X	X	X	
Dental Fear Survey [14]	X	X	X	
Dental Subscale of the Children's Fear Survey Schedule [15]				X^b^
Fear of Pain Questionnaire – Short Form [16]	X	X	X	
Oral Health Impact Profile [17]	X	X	X	
Perceived Stress Scale [18]	X	X	X	
Fatalism Scale [19]	X	X	X	
Health Locus of Control [20]	X	X	X	
Parental Supervision and Involvement [21]	X	X^a^	X^a^	
Family Assessment Measure [22]	X	X	X	
ISEL [23]	X	X^a^		
West Virginia Identity Scale^c^	X	X		
Parental Report Questionnaire [24]		X^b^	X^b^	X^b^

Interviews				
Demographic Interview	X	X^d^	X^a,d^	X^a,b^
Oral Health Interview				X^a,b^
Knowledge and Beliefs about Oral Health	X	X^d^	X^a,d^	
Oral Health Care Utilization	X	X^d^	X^a,d^	
Oral Health History	X	X^d^	X^a,d^	
Oral Health Behaviors	X	X^d^	X^a,d^	
Medical and Family History	X	X^d^	X^a,d^	X^b^
Physical Evaluation	X	X^d^	X^d^	X^d^
Pregnancy History	X	X^d^	X^d^	

*Note*. ^a^youth version; ^b^parental report on children; ^c^used in West Virginia only; ^d^parent and/or researcher assisted.

Other data were collected via interview and covered oral health, medical health, family history, family pedigree, family relationship, and pregnancy history (see Table 1 for the interviews administered to each age group). The pregnancy history was administered to women only and was administered in private. For females under age 18, the pregnancy questionnaire was administered only if the research staff knew there had been a pregnancy. Typically, the parent completed the interviews for him or herself and for all of the children, regardless of their ages, except for the oral health interview for children aged 11 or older. These children completed the oral health interview themselves.

#### Dental Screening and Microbiology Sampling

The clinical protocol was performed in an exam room equipped with a dental chair and dental examination light. Two research staff members (an assistant and either a dentist or dental hygienist) performed standardized periodontal and caries screenings. Participants were instructed not to eat or brush their teeth for at least two hours before the screening. The assessment included documentation of problems with the dentition (tooth loss, caries, restorations, dentures, sealants, plaque, calculus, malocclusion, orthodontic appliances, traumatic injury, erosion, pain), the supporting structures (gingivitis, periodontal destruction, periodontal microbiota, bleeding on probing, *Strep mutans*, pain), and the soft tissue (oral mucosal lesions, malformations, salivary gland function, pain). The oral health screening took approximately 45 minutes. See Table 2 for a summary of the procedures. For children aged 1 – 3, in place of the full dental screening, an abbreviated lift-the-lip exam was conducted to document early childhood caries and tooth loss.

**Table 2:** Components of the dental examination administered by age group

Exam	Children age 1 – 6	Children age 7 – 10	Children age 11 – 17	Adults age 18+
DNA samples	X	X	X	X
Supragingival plaque	X	X	X	X
Tongue scraping	X	X	X	X
Unstimulated saliva secretion rate		X	X	X
Soft tissue exam	X	X	X	X
Denture assessment	X	X	X	X
BANA tongue scraping	X	X	X	X
BANA subgingival plaque		X^a^	X^a^	X
Papillary bleeding score		X^a^	X^a^	X
Periodontal screening^b^				X
Malocclusion^c^		X	X	X
Caries^d^	X	X	X	X
Trauma	X	X	X	X
Throat swab	X	X	X	X

*Note*. ^a^Only if no premedication is needed; ^b^Mean inter- and intra-rater reliabilities 0.77 and 1.00 respectively; ^c^Mean inter- and intra-rater reliabilities 0.75 and 1.00 respectively; ^d^Mean inter- and intra-rater reliabilities 0.83 and 0.98 respectively. Reliabilities were determined using Cohen's kappas.

##### Plaque samples for microbial assessment

Plaque was obtained from supragingival and subgingival tooth surfaces, the tongue, and the throat. Oral microbes from these samples were assessed using BANAMet (OraTec Corp., Manassas, VA), and Dentocult^®^SM Strip mutans (North Bay/Bioscience, Traverse City, MI). Supragingival samples were obtained using a Stimudent^® ^from a caries-free surface. Supragingival samples were also obtained using a scaler from a white spot lesion, an enamel lesion, and a dentin lesion, when these lesions were present. Subgingival plaque was sampled using a curette from the mesial interproximal surfaces of the first molars. If a first molar was missing, the sample was obtained from the next most proximal tooth. The tongue sample was obtained with a Stimudent^®^. The throat sample was obtained using a throat swab. In addition to the semi-quantitative identification of the microbes present in the samples (BANA and Dentocult), the plaque samples were stored for planned DNA analysis.

##### Salivary sampling

Three measures were obtained from saliva. First, saliva was stimulated by having the participant chew on a wax pellet. From this method, a semi-quantitative measure of *Strep mutans *load was obtained using Dentocult^®^SM Strip mutans. Second, unstimulated saliva was used to determine salivary flow rate. To obtain the saliva, subjects ages 4 and older spit into a vial for 3 minutes [3]. Third, from this collected saliva, cotinine levels were then determined using a NicAlert Kit (Nymox Pharmaceutical Corp., Maywood, NJ).

##### Caries assessment

Each coronal tooth surface was assessed for the presence of dental caries and was classified as sound, decayed, filled, or missing. Decayed coronal surfaces were classified using the four level classification method developed by the World Health Organization [4], where decayed surfaces were recorded as D1 for pre-cavitated (e.g., white spot) lesions; D2 for enamel only lesions; D3 for dentinal lesions, and D4 for lesions extending into the pulp. Filled coronal surfaces were classified as filled if any restoration was present on that surface and there was no decay present. Surfaces with both fillings and decay were classified as recurrent decay. Root caries was assessed clinically and classified as present or absent at the tooth level. Tooth loss was measured at the surface level, and all missing surfaces were so marked.

##### Periodontal assessment

A modified version of the Periodontal Screening and Recording (PSR) [5] method was used to evaluate the periodontal status of adults and children older than 17 years of age. The probing depth for the deepest pocket in each sextant of the mouth was obtained. In the case that all teeth in a given sextant were missing, no observation was obtained for that sextant. Thus participants could contribute anywhere from one to six observations. The modified PSR was scored 1 (</= 3.5 mm), 2 (> 3.5 mm but </= 5.5 mm), and 3 (> 5.5 mm). The PSR Index is based on the Community Periodontal Index of Treatment Needs [6], which is endorsed by the World Health Organization (WHO).

Papillary bleeding was assessed using the Loesche Papillary Bleeding Index [7][8]. Up to 26 papillae were assessed per person, depending on the number of teeth.

#### DNA Collection

DNA was collected from biological samples using several approaches. Initially, the first choice was whole blood collected by trained phlebotomists from peripheral veins (approximately 7.5 ml) from all family members aged 1 and older. For family members who were unwilling to provide a blood sample, DNA samples were taken using alternative methods such as saliva sampling, mouthwash sampling or buccal swab sampling. With the availability of improved methodologies, DNA sampling became almost exclusively based on saliva sampling (Oragene*DNA Self-Collection Kit; DNA Genotek Inc., Ottawa, Ontario, Canada).

### Data Processing and Quality Control

Upon completion of a family's clinic visit, the research staff verified the completeness of the data and, rarely, brought back families to complete missing items. Each subject record was assigned a random code number and all personal identifiers (e.g., name, address, etc.) were removed prior to forwarding to the coordinating center. The coordinating center was located at the University of Pittsburgh, and a collaborating site was located at West Virginia University. Biological samples (for DNA and/or microbiota) and water samples were sent to processing and storage sites at the University of Pittsburgh Center for Craniofacial and Dental Genetics and the West Virginia University Research Laboratory of Microbial Pathology and Epidemiology. Hard-copy data were transferred from all field sites to the data management teams at each university via next day mail service. Electronic transfer of data was monitored for completeness by the systems analysts at the University of Pittsburgh. Recruitment logs were sent monthly to the investigators and research program manager for monitoring study progress.

At the coordinating center, interview, dental screening, and physical assessment data were entered into the study's main database initially via hand-entry and subsequently via scannable Teleforms (Cardiff TeleForm, Vista, CA). For the first two years of the study, the self-report data were manually entered into the database. After the self-report data collection was transferred to the PC tablets, the PC tablet data were regularly sent to the coordinating center over a secure network. On an on-going basis, the database manager reviewed the data for inaccuracies and discrepancies, which were reported to the research program manager. The research program manager sent queries to the sites and the data were updated as appropriate. A paper trail of data updates was kept in the participants' records.

Dentocult^®^SM Strip mutans and BANAMet microbiological testing kits were incubated as indicated in the manufacturers' instructions and read by the field staff. Strips were then sent to the study's collaborating microbiology lab in West Virginia for an independent reading and long-term storage. Lab and site readings were compared for quality control. Plaque samples, throat swabs, and saliva samples were also sent to the collaborating microbiology lab in West Virginia for long-term storage. Quality control checks were performed on the plaque samples and throat swabs.

To insure that the standards and protocols were being followed properly on site, one of the West Virginia co-investigators accompanied the research staff to satellite clinics in West Virginia bi-monthly, and the Pennsylvania co-investigator visited the clinics in Pennsylvania. During these visits, the co-investigators observed the dental screenings, made suggestions as needed, and answered staff questions. The research program manager visited the Pennsylvania sites one to two times per year, as well. The principal investigator monitored the quality of the oral microbial samples with regular calls to the labs and periodic on-site audits. Further, there were weekly conference calls between the investigators and research staff from each site to address study issues.

#### Calibration

Calibration sessions for both the caries and periodontal screenings occurred prior to the initiation of the study and periodically during the four years of data collection. At the calibration sessions, first a review of the techniques for caries and periodontal disease data collection was provided to the examiners. The review was followed by testing on participants to determine the inter- and intra-examiner reliability of the examiners. Inter-rater reliability was determined by comparing assessments performed by each examiner/recorder team against the assessment from the gold standard examiner/recorder team. Three sections of the clinical protocol were calibrated in this manner, the caries, periodontal, malocclusion assessments. To perform the periodontal assessment and recording, the examiners used one manual calibrated probe. Intra-rater reliability was determined by comparing assessments performed by each examiner/recorder team on the same participant on two consecutive days. At each calibration session, screenings were performed on two children with caries, two adolescents with caries, and two adults with attachment loss that included periodontal pockets of at least 5 mm from the base of the pocket to the free gingival margin. See Note in Table 2 for the inter- and intra-rater reliabilities for the components of the screening.

### Power

It is difficult to provide a single power calculation for such a complex and multi-faceted project. As an illustration of the power of this study design, we estimated the power of this study sample for our proposed genetic studies. Because it is impossible to provide exact power calculations, we estimated the power as a function of a range of disease allele frequencies and genotype relative risks (GRR) using the Genetic Power Calculator [9] for a simple chi-squared test of allelic association assuming an r^2 ^of 0.8, a multiplicative model, and a population disease prevalence equal to that observed in the families collected to date for two sample phenotypes: "any dental caries in the permanent dentition," and "no dental caries in the permanent dentition." We assumed an alpha level of 0.001 and performed the calculations to estimate the power to detect genetic association in a genome-wide study design.

**Figure 1:**
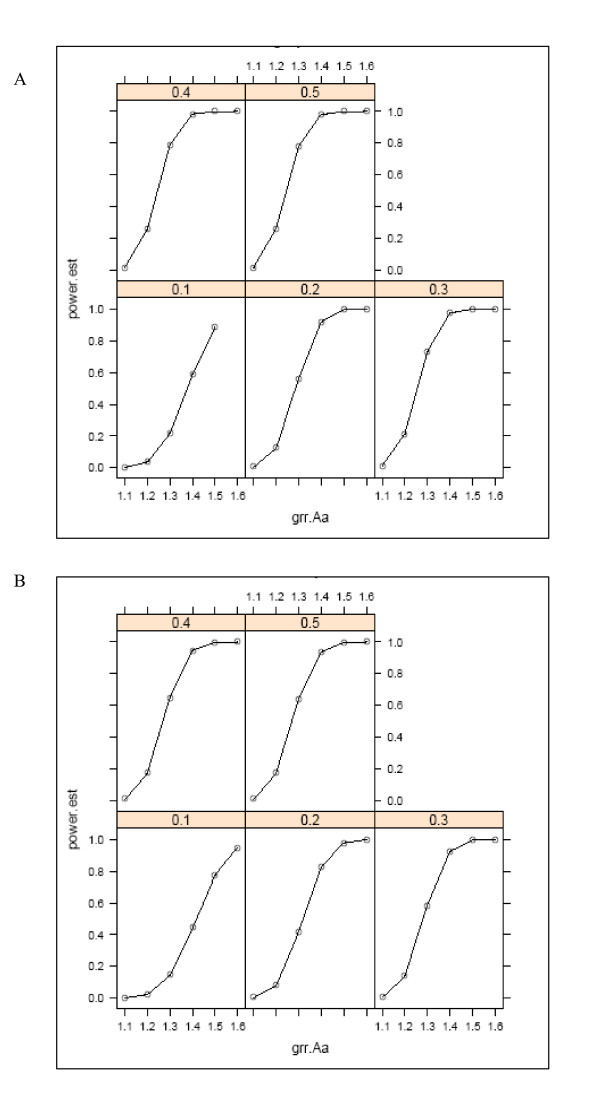
**A**. **Power to detect genetic association as a function of genotype relative risk (GRR) and disease allele frequency, at a significance level of 0.001, for the phenotype of "any dental caries in the permanent dentition".B**. **Power to detect genetic association as a function of genotype relative risk (GRR) and disease allele frequency, at a significance level of 0.001, for the phenotype of "no dental caries in the permanent dentition".**

As can be seen in Figure 1A and 1B, the power of our sample to detect genetic association is very high if the disease allele frequency is greater than about 0.2 and the GRR is greater than about 1.4.

## Discussion

In this study, families were recruited from six sites across two states in northern Appalachia and invited to participate in an etiology study of their oral health. This report describes the recruitment strategies and study protocol.

With data from our study, we will be able to address many different types of hypotheses. For example, we will be able to examine the degree to which factors at a given level contribute to oral health status, such as whether tooth brushing, flossing, and a low sugar diet (individual level behaviors) are associated with less caries. In addition, we will be able to examine cross-level interactions. For example, tooth brushing (individual level behavior) and pit and fissure genes (genetics) may interact such that persons with poor brushing behavior and genes encoding deeper pits and fissures are at greater risk than persons with either risk factor by itself or the additive risk of the two risk factors. Furthermore, we will be able to examine more complex mediating pathways that cross multiple levels. For example, the pathway through which socioeconomic status (family level factor) is associated with caries could include brushing (individual level behavior), salivary flow (biologic factor) and microbial population (microbes). Finally, our hypotheses will not be limited to humans, because we will have access to genetic information about microbes as well.

Although there are many strengths to our study, there are weaknesses as well. For example, our study sample is drawn from volunteers; it is not a true random sample. However, this problem is mitigated by the approach of the current study in developing models that could be tested in future studies with true random samples. Such studies will be needed to evaluate the generalizability of our models. In addition, as yet we have no comparison group undergoing the same protocol. Thus we will be unable to address how our sample differs from non-Appalachians. To mitigate this weakness to some extent, we will be able to compare some of our data elements to nationally available data, and we are developing collaborations with other USA and non-USA cohort studies.

In sum, the aim of COHRA is to determine genetic, microbial, individual, family, and community factors that contribute to poor oral health status in Appalachia. We believe that there are trajectories of oral and systemic health and that an individual's trajectory may be determined early in life. Thus, we are particularly interested in the contributors to the oral health status of children. Our protocol is designed to examine factors hypothesized to contribute to children's oral health status at multiple levels [10]. COHRA applies a family-based approach to the study of social and health-related factors impacting the oral health of children in Appalachia.

## Competing interests

The authors declare that they have no competing interests.

## Authors' contributions

DEP drafted the manuscript. RJW, RJC, DWM, RET, JGT, and MLM made substantial contributions to conception and design of the study and acquisition and analysis and interpretation of the data, were involved in revising the manuscript, and gave final approval for publication.

## Pre-publication history

The pre-publication history for this paper can be accessed here:


